# STING Restricts EV-A71 Infection by Regulating T Cell Development and Enhancing Immune Cell Effector Function

**DOI:** 10.3390/ijms262311441

**Published:** 2025-11-26

**Authors:** Huiqiang Wang, Ya Wang, Shuo Wu, Lijun Qiao, Wen Sheng, Haiyan Yan, Kun Wang, Ge Yang, Jiandong Jiang, Yuhuan Li

**Affiliations:** 1 CAMS Key Laboratory of Antiviral Drug Research, Beijing Key Laboratory of Technology and Application for Anti-Infective New Drugs Research and Development, NHC Key Laboratory of Biotechnology of Antibiotics, Institute of Medicinal Biotechnology, Chinese Academy of Medical Sciences and Peking Union Medical College, Beijing 100050, China; wangya@imb.pumc.edu.cn (Y.W.); wushuo@imb.pumc.edu.cn (S.W.); qiaolijun@imb.pumc.edu.cn (L.Q.); shengwen@imb.pumc.edu.cn (W.S.); yanhaiyan@imb.pumc.edu.cn (H.Y.); wangkun@imb.pumc.edu.cn (K.W.); yangge@imb.pumc.edu.cn (G.Y.); jiangjiandong@imb.pumc.edu.cn (J.J.); 2 Division for Medicinal Microorganism-Related Strains, CAMS Collection Center of Pathogenic Microorganisms, Chinese Academy of Medical Sciences and Peking Union Medical College, Beijing 100050, China; 3 State Key Laboratory of Bioactive Substances and Functions of Natural Medicines, Institute of Medicinal Biotechnology, Chinese Academy of Medical Sciences and Peking Union Medical College, Beijing 100050, China

**Keywords:** STING, diABZI, EV-A71, immune regulation, inflammation

## Abstract

Previous studies have reported that Enterovirus A71 (EV-A71) infection could activate STING-related signaling pathways in vitro, but the role of STING in EV-A71 infection in vivo and the associated immune regulatory mechanisms remain unclear. Here, we used the STING-specific agonist diABZI to activate STING and STING-knockout mice to jointly study the role and mechanism of regulating STING on EV-A71 infection in vivo. The results showed that activating STING could inhibit the in vivo replication of EV-A71, alleviate clinical symptoms in infected mice, and increase the survival rate. Conversely, STING knockout significantly promoted viral replication in vivo and increased the lethality and severity of EV-A71 infection. Mechanistic studies further revealed that STING activation exerts its antiviral effects by stimulating interferon signaling pathways, upregulating the expression of interferon-stimulated genes (ISGs). Additionally, STING activation also modulated the serum cytokine response profile. Moreover, STING activation drove the expansion of diverse immune cell populations, including T cells, natural killer (NK) cells and myeloid cells. In contrast, STING knockout not only reduced the proportion of thymic T cells and impeded T cell developmental progression from double-positive (DP) to single-positive (SP) stages, but also impaired the effector functions of CD8+ T cells and NK cells during viral infection. In summary, this study demonstrates that STING activation effectively suppresses EV-A71 replication and mitigates infection symptoms by modulating immune and inflammatory responses. These findings provide a foundational framework for understanding how STING coordinates antiviral immunity and inform future investigations into STING-targeted therapies for viral infections.

## 1. Introduction

The innate immune system is the first line of defense against pathogenic microbial infections, detecting microbial infections by sensing microbial molecules or host signaling molecules released from damaged host cells. For example, after virus infection, pattern recognition receptors (PRRs) such as Toll-like receptors (TLRs), cytoplasmic NOD-like receptors (NLRs), intracellular retinoic acid-inducible gene-I-like receptors (RLRs), and cyclic GMP-AMP synthase (cGAS) in host cells recognize the dsRNA or DNA of the viruses, and the antiviral natural immune response pathway is activated to resist virus invasion [1,2,3,4,5]. Therefore, PPRs are the “sentinels” and “scouts” of the immune system. By identifying pathogens, they initiate immune responses at the first moment, forming the cornerstone of the body’s anti-infection immunity and inflammatory responses. PPRs also provides the necessary signals for the activation of adaptive immunity by guiding the function of antigen-presenting cells, influencing the intensity, type and duration of immune responses.

Stimulator of interferon genes (STING, also named MITA, MPYS or ERIS) is a signal transduction molecule found in recent years that is closely related to the innate immune response [6]. The cGAS-STING pathway can mediate the recognition of viruses and activate the corresponding cellular defense mechanism, including the activation of NF-κB pathway and interferon expression [6,7]. First, cytosolic DNA induces the synthesis of cGAMP after binding to cGAS, and cGAMP induces the transfer of STING from the endoplasmic reticulum to the Golgi apparatus, resulting in allosteric and polymerization. The polymerized STING recruits and activates TBK1, which further induces the dimerization and activation of the transcription factor IRF3, and finally realizes the transcription of interferon genes [8]. Thus, it is possible to establish an immune state in immune and non-immune cells through cGAS-STING-IRF3 signal transduction, thereby limiting microbial infections such as viruses.

Although the function of the cGAS/STING signaling axis in innate immune responses to DNA viruses has been well deciphered, there is increasing evidence that it has an equally important contribution in controlling RNA virus infection [9,10,11,12,13,14,15]. Previous studies have shown that STING is involved in the regulation of multiple RNA virus infections, including coronavirus, Dengue virus (DENV), Zika virus (ZIKV), human immunodeficiency virus (HIV), hepatitis C (HCV), and enterovirus A71 (EV-A71) [11,12,13,14,15].

EV-A71, belonging to the genus Enterovirus of the *Picornaviridae* family, can cause hand, foot and mouth disease (HFMD), and a few infections can cause aseptic meningitis, brainstem encephalitis, polio-like paralysis, and even death [16,17]. Recent studies have shown that the levels of factors such as IL-10, IL-13, IP-10, MCP-1, MIG, and G-CSF in the blood and cerebrospinal fluid of patients infected with EV-A71 increase significantly [18,19]. In addition, the levels of inflammatory factors such as IL-2, IL-6 and G-CSF in critically ill children are higher than those in ordinary children, indicating that EV-A71 infection can cause severe stress and systemic inflammatory responses in the body [20].

Studies have reported that treatment with the TLR7 agonists R837 and GS-9620 can inhibit the in vivo replication of EV-A71 through regulating the immune response [21,22]. AY9944 exerts an anti-enterovirus effect by inhibiting 7-dehydrocholesterol reductase to activate the IRF-3-regulated interferon response [23]. In addition, many natural products can exert anti-EV-A71 effects by regulating inflammatory responses [24]. These studies indicate that the infection of EV-A71 can be controlled by regulating the immune and inflammatory responses of the body.

Two independent studies have reported that EV-A71 infection can activate STING-related signaling pathways in vitro, and that viral 2C and 2A proteins can regulate STING protein levels during infection, thereby limiting STING-related immune responses [5,15]. However, the effect of STING on EV-A71 infection in vivo and the changes and roles of the related immunity regulated by STING in EV-A71 infection remain unclear. In this study, we investigated the role and mechanism of regulating STING on EV-A71 infection through STING agonist activation and STING knockout in vivo, which may lead to significant discoveries in understanding the pathogenesis of EV-A71 and treatment of EV-A71 infection.

## 2. Results

### 2.1. STING Activation Improves Survival Rates and Relieves Clinical Symptoms of EV-A71-Infected Mice

**Figure 1:**
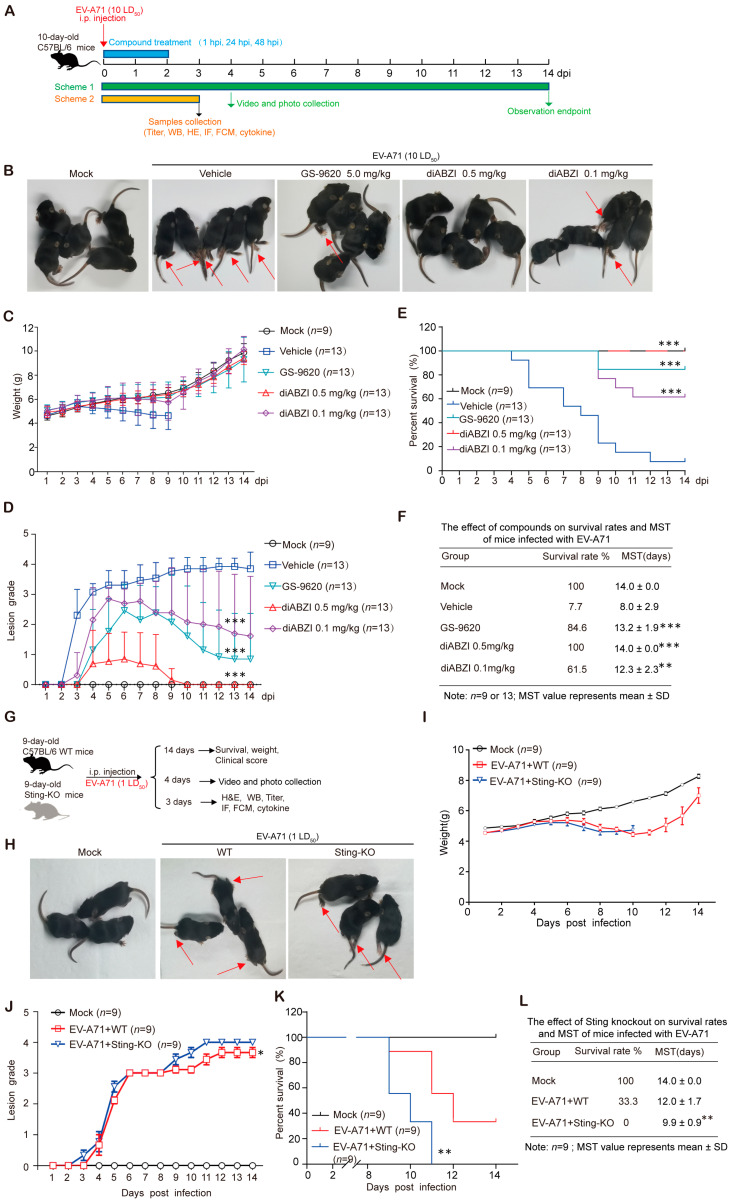
Regulation of STING affects EV-A71 infection in mice. (**A**) Schematic presentation of animal experiment design. Ten-day-old C57BL/6J mice were intraperitoneally inoculated with 10 LD_50_ of EV-A71-H-MA. One hour after virus infection, the mice were treated with diABZI or GS-9620 once daily for 3 days. (**B**) Representative photographs of mice at 4 dpi and the arrow points to the weak or paralyzed hind limb (*n* = 5). (**C**) Daily body weight monitoring of mice (*n* = 9 or 13). (**D**–**F**) Clinical scores (**D**) and survival kinetics (**E**,**F**) of mice over 14 days (*n* = 9 or 13), *** *p* < 0.001 by Ridit assay (D), ** *p* < 0.01, *** *p* < 0.001 by a Log-Rank (Mantel–Cox) assay (E,F). (**G**) Schematic presentation of animal experiment design. Nine-day-old WT mice and Sting-KO mice were intraperitoneally inoculated with 1 LD_50_ of EV-A71-H-MA. (**H**) Representative photographs of mice at 4 dpi and the arrow points to the weak or paralyzed hind limb (*n* = 3). (**I**) Daily body weight monitoring of mice (*n* = 9). (**J**–**L**) Survival kinetics (**K**,**L**) and clinical scores (**J**) of mice over 14 days (*n* = 9). ** *p* < 0.01 by a Log-Rank (Mantel–Cox) assay (**K**,**L**) and * *p* < 0.05, by Ridit assay (**J**). Data are from one experiment with *n* = 9 or 13 mice per group.

To investigate the potential anti-EV-A71 activity of STING signaling in vivo, we first evaluated the therapeutic effect of the specific STING agonist diABZI in a mouse model. For comparison, GS-9620, a known Toll-like receptor 7 (TLR7) agonist with reported efficacy against EV-A71, was included as a positive control [22]. Ten-day-old C57BL/6J mice were intraperitoneally challenged with a lethal dose (10 LD_50_) of EV-A71-H-MA. Treatment with diABZI or GS-9620 was initiated 1 h post-infection and administered once daily for 3 days (Figure 1A).

Both diABZI and GS-9620 treatments markedly ameliorated disease progression compared to the vehicle control. Specifically, they significantly reduced the incidence of hind limb paralysis at 4 dpi (Figure 1B and Supplementary Video S1), mitigated virus-induced weight loss (Figure 1C), and improved clinical scores (Figure 1D). Most importantly, both agonists conferred significant protection against lethality, resulting in markedly enhanced survival rates (Figure 1E). The mean survival time (MST) of the vehicle-treated control mice was 8.0 ± 2.9 days (Figure 1F). In contrast, treatment with 0.5 mg/kg or 0.1 mg/kg diABZI significantly extended the MST to 14.0 ± 0 days and 12.3 ± 2.3 days, respectively (Figure 1F). Similarly, GS-9620 treatment yielded an MST of 13.2 ± 1.9 days (Figure 1F). These findings demonstrate that pharmacological activation of STING, akin to TLR7 activation, provides a substantial protective effect against lethal EV-A71 challenge in mice.

### 2.2. STING Knockout Significantly Enhances the Lethality and Exacerbates Severity of EV-A71 Infection in Mice

To further investigate the role of STING in combating EV-A71 infection in vivo, we employed homozygous STING-knockout C57BL/6J mice (designated Sting-KO), with the experimental timeline outlined in Figure 1G. Nine-day-old WT and Sting-KO littermates were inoculated intraperitoneally with a sublethal dose (1 LD_50_) of EV-A71-H-MA. STING deficiency markedly exacerbated disease severity, leading to significantly more severe hind-limb paralysis at 4 dpi compared to WT mice infected with EV-A71 (Figure 1H,I and Supplementary Video S2). Although body weight changes were not significantly different between the groups (Figure 1I), Sting-KO mice presented substantially higher clinical scores following viral challenge (Figure 1J). Crucially, STING knockout resulted in significantly increased mortality (Figure 1K). The mean survival time (MST) of WT mice infected with EV-A71 was 12.0 ± 1.7 days, whereas Sting-KO mice infected with EV-A71succumbed significantly earlier, with an MST of 9.9 ± 0.9 days (Figure 1L). In summary, genetic ablation of STING significantly sensitizes mice to EV-A71 infection, worsening clinical disease and increasing lethality. These findings provide direct genetic evidence that an intact STING signaling pathway is essential for host defense against EV-A71 in vivo.

### 2.3. Regulation of STING Affects the Replication of EV-A71 in Mice

**Figure 2:**
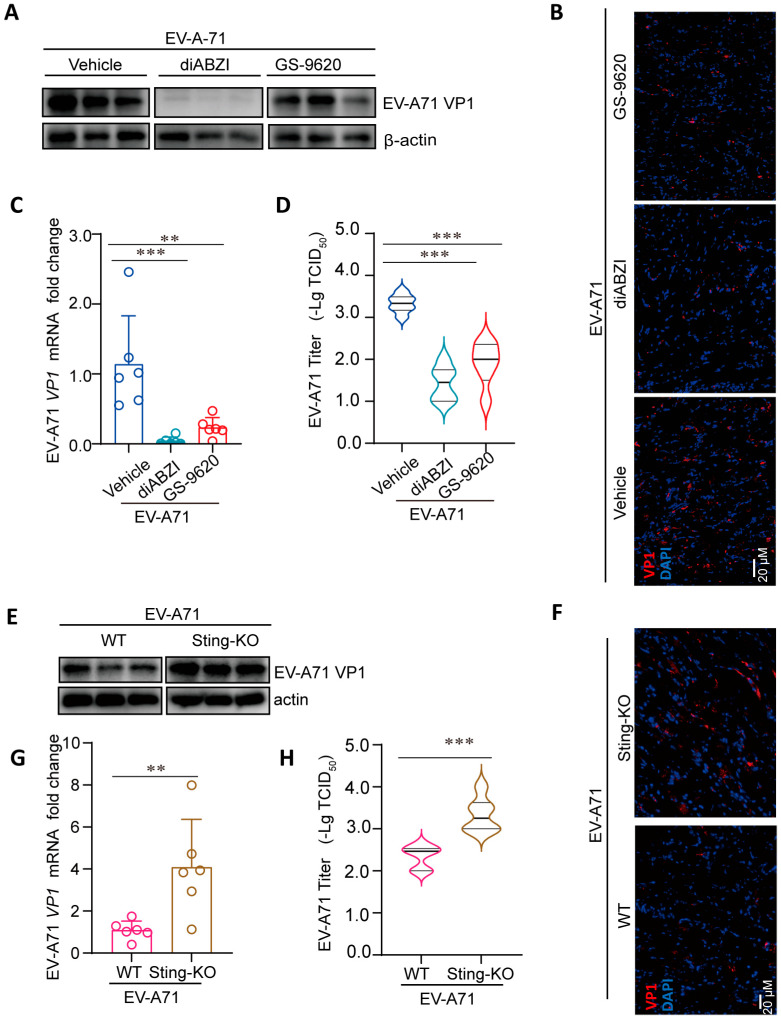
Regulation of STING affects the replication of EV-A71 in mice. (**A**–**D**) Ten-day-old C57BL/6J mice were intraperitoneally inoculated with 10 LD_50_ of EV-A71-H-MA. One hour after virus infection, the mice were treated with diABZI or GS-9620 once daily for 3 days. Muscle tissues were detected by Western blot assay or IF assay with indicated antibodies ((**A**,**B**), *n* = 3), qRT-PCR assay with specific primers ((**C**), *n* = 6) and viral titer assays ((**D**), *n* = 6). ** *p* < 0.01, *** *p* < 0.001, one-way ANOVA with Holm–Sidak multiple comparisons test (**C**,**D**). (**E**,**F**) Nine-day-old WT mice and Sting-KO mice were intraperitoneally inoculated with 1 LD_50_ of EV-A71-H-MA. Muscle tissues were detected by Western blot assay or IF assay with indicated antibodies ((**E**,**F**), *n* = 3), qRT-PCR assay with specific primers ((**G**), *n* = 6) and viral titer assays ((**H**), *n* = 6), ** *p* < 0.01, *** *p* < 0.001, two-tailed Student’s *t*-test (**G**,**H**). Data are from one experiment with *n* = 3 or 6 mice per group.

To delineate the role of STING signaling in controlling EV-A71 replication in vivo, we quantified viral loads in the primary site of infection. Consistent with prior findings that hind-limb muscles support robust EV-A71 replication, we analyzed muscle tissues harvested at 3 dpi from infected mice treated with diABZI, GS-9620, or vehicle [25]. Pharmacological activation of STING with diABZI, as well as TLR7 activation with GS-9620, significantly suppressed viral replication. This was evidenced by marked reductions in EV-A71 VP1 protein (Figure 2A,B) and viral RNA levels (Figure 2C) in muscle tissues. Concordantly, viral titers were substantially decreased by approximately 2 log (diABZI) and 1 log (GS-9620), respectively (Figure 2D). These data demonstrate that STING activation potently restricts EV-A71 replication in vivo. Conversely, Sting-KO mice exhibited significantly elevated levels of VP1 protein (Figure 2E,F) and viral RNA (Figure 2G) compared to wild-type controls. This enhanced viral replication was further confirmed by an approximately 1 log increase in viral titers in the muscles of Sting-KO mice (Figure 2H). Collectively, these results establish that the STING pathway plays a critical role in limiting EV-A71 replication in vivo.

### 2.4. STING Affects Viral Replication Through the Regulation of Immunity and Inflammation

**Figure 3:**
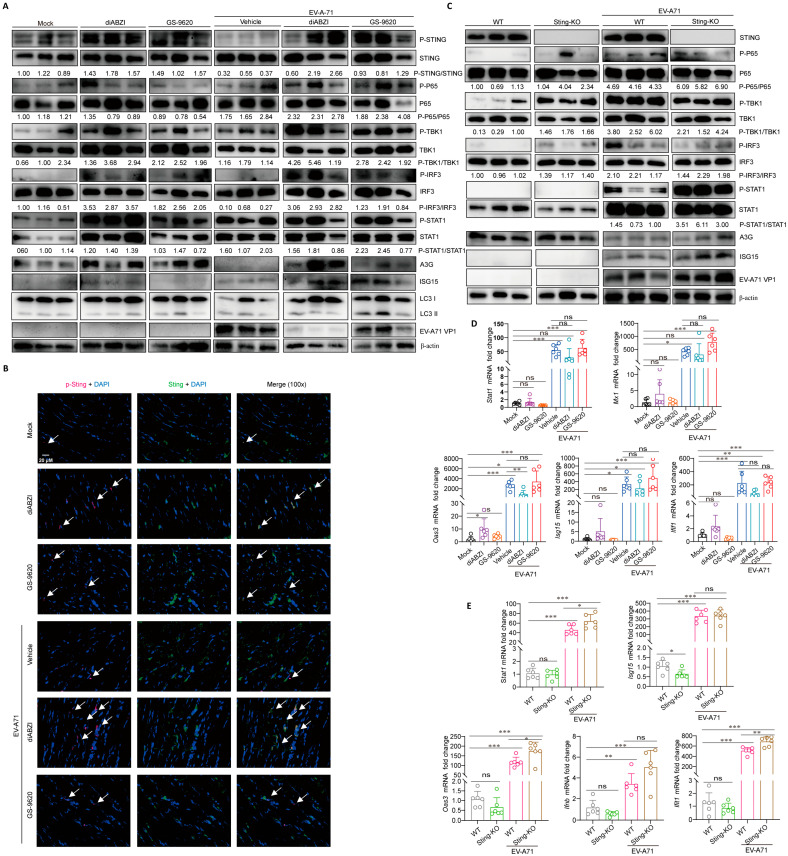
The influence of STING regulation on its related signaling pathways and ISGs expression. Ten-day-old C57BL/6J mice were intraperitoneally inoculated with 10 LD_50_ of EV-A71-H-MA. One hour after virus infection, the mice were treated with diABZI or GS-9620 once daily for 3 days. Muscles were detected by Western blot assay ((**A**), *n* = 3), IF assay (**B**), or qRT-PCR assay ((**D**), *n* = 6). The white arrow represents the phosphorylated STING. Nine-day-old WT mice and Sting-KO mice were intraperitoneally inoculated with 1 LD_50_ of EV-A71-H-MA. Muscles were detected by Western blot assay ((**C**), *n* = 3) or qRT-PCR assay ((**E**), *n* = 6). Data are from one experiment with *n* = 3 or 6 mice per group. * *p* < 0.05, ** *p* < 0.01, *** *p* < 0.001, one-way ANOVA with Holm–Sidak multiple comparisons test or two-tailed Student’s *t*-test, and “ns” indicates no significant difference (**D**,**E**).

To explore the mechanism through which STING affects the replication and infection of EV-A71 in vivo, we examined changes of pathway proteins related to STING in the muscles, including NF-κB, autophagy and the interferon signaling pathway [4,8,12]. In both EV-A71 infection and non-infection conditions, diABZI treatment can stimulate the phosphorylation of STING, TBK1, IRF3, and STAT1 in muscles, and upregulate the expression of interferon stimulated genes (ISGs), including APOBEC3G (A3G) and interferon-stimulated gene 15 (ISG15), but has no significant effect on the phosphorylation of P65 and LC3 conversion (LC3-I to LC3-II) (Figure 3A,B). Similar to the protein results, mRNA detection results showed that in the absence of viral infection, diABZI treatment significantly upregulated the mRNA level of 2,5′-oligoadenylate synthase 3 (Oas3) (Figure 3D). In the case of viral infection, the mRNA levels of Stat1, tetratricopeptide repeats 1 (Ifit1), myxovirus resistance protein 1 (Mx1), Oas3, and Isg15 are upregulated by EV-A71 infection, while only Oas3 is significantly downregulated after diABZI treatment, which may be due to the inhibition of viral replication after diABZI treatment, thereby reducing the stimulating expression of Oas3 (Figure 3D). These results suggest that STING activation in mice exerts anti-EV-A71 effects by interferon signaling pathways, rather than by regulating autophagy. In addition, consistent with our previous in vitro studies, EV-A71 infection downregulates A3G protein levels, but has no effect on ISG15 (Figure 3A) [26]. Interestingly, GS-9620, as a TLR7 agonist, can also stimulate the phosphorylation of STING and downstream proteins in muscles and upregulate the expression of A3G and ISG15 (Figure 3A). These results suggest that the STING and TLR signaling pathways may have interactive regulatory effects.

Interestingly, the results showed that STING knockout had no effect on the phosphorylation of P65, TBK1, and IRF3 stimulated by EV-A71 infection (Figure 3C), indicating that other PRR signaling pathways may compensatively compensate for the effects of STING knockout on IFN signaling pathway. Moreover, compared with WT mice infected with EV-A71, the enhanced replication of EV-A71 in Sting-KO mice instead stimulated higher protein levels of P-STAT1 and ISG15 (Figure 3C). In the absence of viral infection, STING knockout had certain downregulation on the mRNA level of Isg15 (Figure 3E). However, due to the enhanced replication of EV-A71, the mRNA levels of Stat1, Oas3, and Ifit1 after STING knockout were slightly higher than those in WT mice infected with EV-A71 (Figure 3E).

Studies have shown that tissue damage induced by EV-A71 infection, coupled with the production of proinflammatory cytokines/chemokines, represents a critical driver of severe disease and even death [27,28]. DiABZI and GS-9620 treatment mitigated EV-A71 induced pathological injury, while STING knockout aggravated the damage, as evidenced by the H&E staining of muscle sections (Supplementary Figure S1A,B). In line with this, the results showed that diABZI treatment reduced the mRNA levels of inflammatory factor IL-6 and Ip-10, but the knockout of STING did not significantly enhance the mRNA levels of IL-6 and Ip-10 in EV-A71-infected mice. (Supplementary Figure S1C,D). Furthermore, although the knockout of STING itself does not affect the expression of IL-6 and Ip-10, the absence of STING leading to uncontrolled viral replication may maintain high levels of inflammatory cytokines. This means that this high level of inflammatory cytokines is due to the persistent infection of the virus rather than being directly regulated by STING.

**Figure 4:**
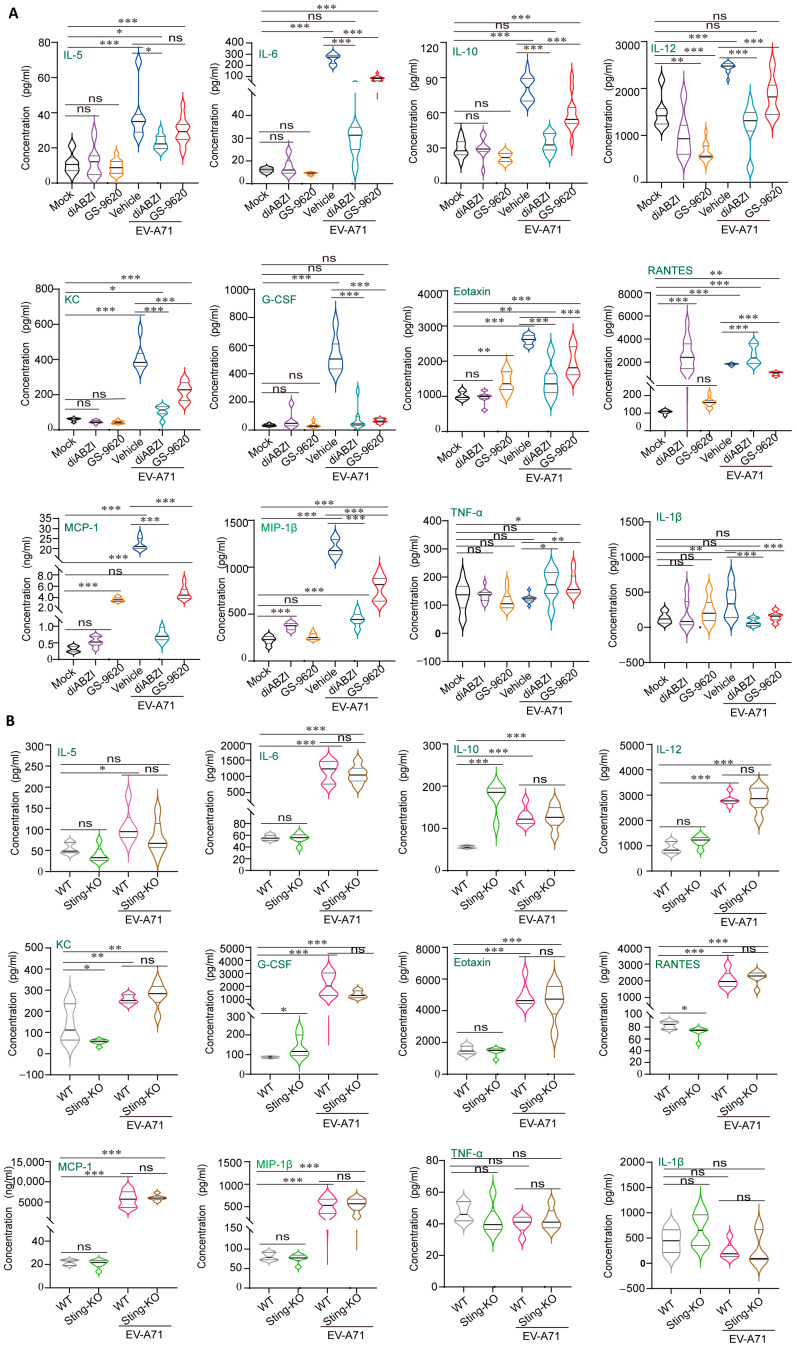
The influence of STING regulation on cytokine/chemokine levels in serum under EV-A71 infection and non-infection conditions. (**A**) Ten-day-old C57BL/6J mice were intraperitoneally inoculated with 10 LD_50_ of EV-A71-H-MA. One hour after virus infection, the mice were treated with diABZI or GS-9620 once daily for 3 days. Serum cytokine/chemokine expression was analyzed via Bio-Plex Pro^TM^ Assays (*n* = 6). (**B**) Nine-day-old WT mice and Sting-KO mice were intraperitoneally inoculated with 1 LD_50_ of EV-A71-H-MA, and serum cytokine/chemokine expression was analyzed via Bio-Plex Pro^TM^ Assays (*n* = 6). Data are from one experiment with *n* = 6 mice per group. * *p* < 0.05, ** *p* < 0.01, *** *p* < 0.001, one-way ANOVA with Holm–Sidak multiple comparisons test or two-tailed Student’s *t*-test, and “ns” indicates no significant difference (**A**,**B**).

Serum cytokine/chemokine profiling revealed that EV-A71 infection did increase the concentrations of important pro-inflammatory cytokines/chemokines. Under non-infectious conditions, treatment with diABZI and GS-9620 had no significant effect on the levels of IL-5, IL-6, IL-1β, TNF-α, IL-10, KC, and G-CSF in serum, but downregulated the level of IL-12 (Figure 4A). In addition, we found that the levels of RANTES and MIP-1β were upregulated after diABZI treatment, while the levels of MCP-1 and Eotaxin were upregulated after GS-9620 treatment, suggesting the difference between STING and TLR pathways in immune regulation (Figure 4A). In addition, under non-infectious conditions, knockout of STING upregulated the levels of IL-10 and G-CSF, downregulated the levels of KC and RANTES, and had no significant effect on other cytokines/chemokines (Figure 4B). Comprehensively considering the factor changes after STING activation and knockout, we found that the level changes of RANTES showed exactly opposite trends, suggesting that there may be a relatively close connection between RANTES and STING, which is worthy of further in-depth research in the future.

Under the condition of viral infection, EV-A71 infection itself significantly upregulated the levels of cytokines/chemokines, and the upregulation effect was much greater than the upregulation changes of some factors caused by diABZI and GS-9620 treatment (Figure 4A). Therefore, compared with the vehicle group, the levels of cytokines/chemokines decreased significantly after diABZI and GS-9620 reduced EV-A71 replication (Figure 4A). Moreover, since the antiviral effect of 0.5 mg/kg diABZI was superior to that of 5 mg/kg GS-9620, the downregulation of cytokine/chemokine levels in the diABZI treatment group was more significant. Interestingly, although the replication of EV-A71 was enhanced after STING knockout, there was no significant difference in cytokine/chemokine levels compared with WT mice infected with EV-A71 (Figure 4B). This might be because the levels of cytokines/chemokines change dynamically with the time of viral infection, but we only detected one time point after infection. In conclusion, our present results suggest that STING exerts antiviral effects through the regulation of immunity and inflammation.

### 2.5. The Effect of STING on the Composition and Function of Immune Cells in the Spleen

**Figure 5:**
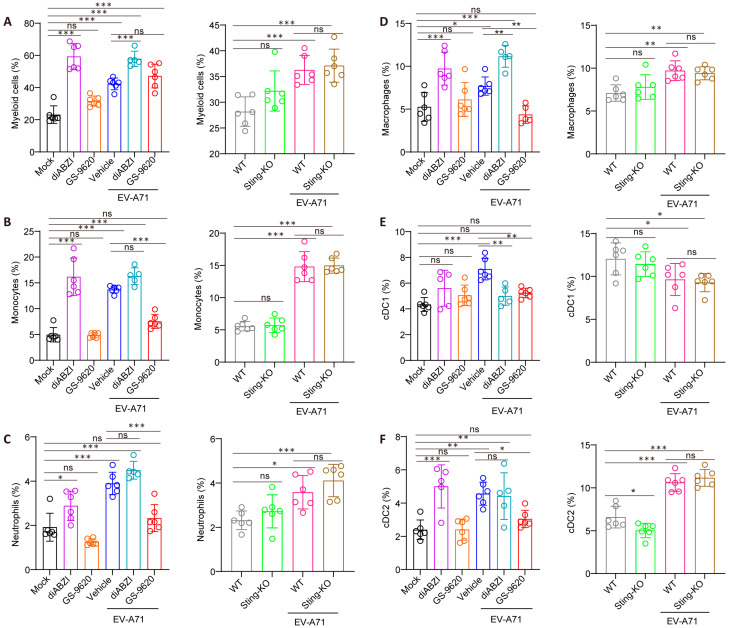
Modulation of splenic myeloid cell populations by STING. The percentages of total myeloid cells (**A**), monocytes (**B**), neutrophils (**C**), macrophages (**D**), and distinct dendritic cell (**C**,**D**) subsets (**E**,**F**) were analyzed via flow cytometry (*n* = 5 or 6). Data are from one experiment with *n* = 6 mice per group. * *p* < 0.05, ** *p* < 0.01, *** *p* < 0.001, one-way ANOVA with Holm–Sidak multiple comparisons test or two-tailed Student’s *t*-test, and “ns” indicates no significant difference.

As the largest secondary lymphoid organ, the spleen engages a diverse repertoire of pattern recognition receptors (PRRs) upon recognizing infection, which subsequently elicit the activation of immune cells, secretion of cytokines, and clearance of pathogens by phagocytes [29]. Therefore, we conducted a further investigation into the impacts of STING on the composition and functions of diverse immune cell types within the spleen. Unlike GS-9620, diABZI treatment specifically elevates the percentage and number of splenic myeloid cells, including monocytes, neutrophils, macrophages, and dendritic cells (DCs) (Figure 5 and Supplementary Figure S2A–F). Furthermore, beyond such elevation in immune cell proportions, researchers have also reported that STING activation can induce macrophage reprogramming toward M1 polarization, promote DC maturation, and enhance their antigen-presenting capacity [30,31,32]. In contrast, STING knockout had no significant effect on the number and proportion of cells in these splenic myeloid cell subsets, except for macrophages and cDC2 (Figure 5 and Supplementary Figure S2A–F).

**Figure 6:**
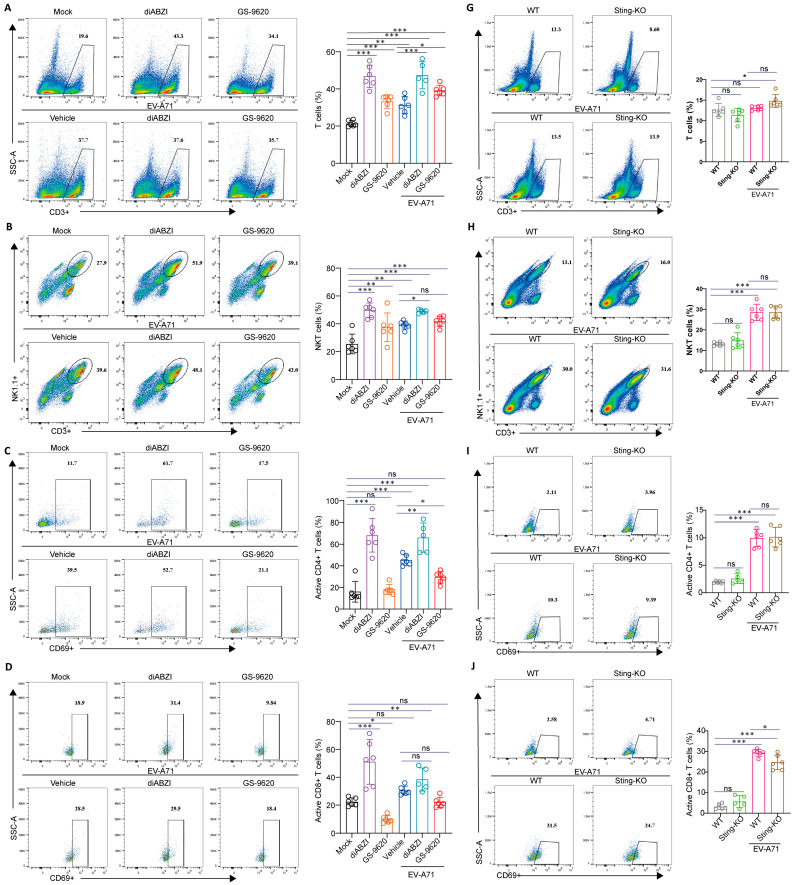

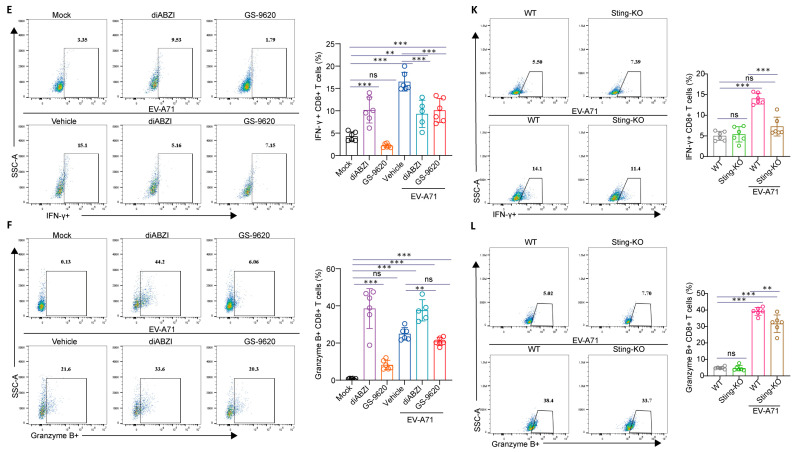
Effect of STING on splenic T cells. (**A**–**F**) Ten-day-old C57BL/6J mice were intraperitoneally inoculated with 10 LD_50_ of EV-A71-H-MA. One hour after virus infection, the mice were treated with diABZI or GS-9620 once daily for 3 days. The percentages of different types of T cells in spleen were measured by flow cytometry (*n* = 5 or 6). (**G**–**L**) Nine-day-old C57BL/6J WT mice and Sting-KO mice were intraperitoneally inoculated with 1 LD_50_ of EV-A71-H-MA. The percentages of different types of T cells in spleen were measured by flow cytometry (*n* = 5 or 6). Data are from one experiment with *n* = 6 mice per group. The color gradient from blue to red indicates a progressive increase in cell density. Circle and wireframe represent flow cytometry gating. * *p* < 0.05, ** *p* < 0.01, *** *p* < 0.001, one-way ANOVA with Holm–Sidak multiple comparisons test or two-tailed Student’s *t*-test, and “ns” indicates no significant difference.

Subsequently, we explored the impact of STING on T and natural killer (NK) cell populations. Interestingly, in the present study, the administration of diABZI not only elevated the number and proportions of total T cells (Figure 6A and Supplementary Figure S3A) and natural killer T (NKT) cells (Figure 6B and Supplementary Figure S3B), but also activated a higher proportion of CD4+ and CD8+ T cells (Figure 6C,D and Supplementary Figure S3C,D). Notably, in comparison to the control group, the CD8+ T cells became functionally superior following diABZI treatment, with significant augmentation of their capacity to secrete IFN–γ and granzyme B (Figure 6E,F and Supplementary Figure S3E,F), which is crucial in virus clearance. Additionally, diABZI enhanced the secretory capacity of NK cells for Granzyme B^+^, IFN-γ^+^, and TNF-α^+^, reflecting improved effector functions (Supplementary Figure S4A–C). Although less pronounced than diABZI, GS-9620 treatment also upregulated T and NKT cell frequencies, and augmented NK cell effector functions. Importantly, although the number of T cells decreased to some extent after STING knockout, no significant alterations were observed in the proportions of T and NKT cells (Figure 6G,H and Supplementary Figure S3G,H). Basal CD8+ T cell and NK cell responsiveness remained unchanged under uninfected conditions. However, the activation and secretory capabilities of CD8+ T cells were markedly diminished in the face of viral infection, indicating impaired function of CD8+ T cells (Figure 6J–L and Supplementary Figure S3J–L). Furthermore, STING knockout also led to a decrease in the secretory function of NK cells during viral infection (Supplementary Figure S4D–F). These findings suggest that STING is critical for maintaining the effector functions of innate immune cells, and the impairment of such functions following STING knockout may contribute to increased viral susceptibility. Collectively, these results demonstrate that STING activation promotes the expansion of multiple immune cell populations, whereas STING deficiency does not alter baseline cell frequencies but impairs their ability to mount effective responses during viral infection.

### 2.6. The Impact of STING on T-Cell Development in the Thymus

**Figure 7:**
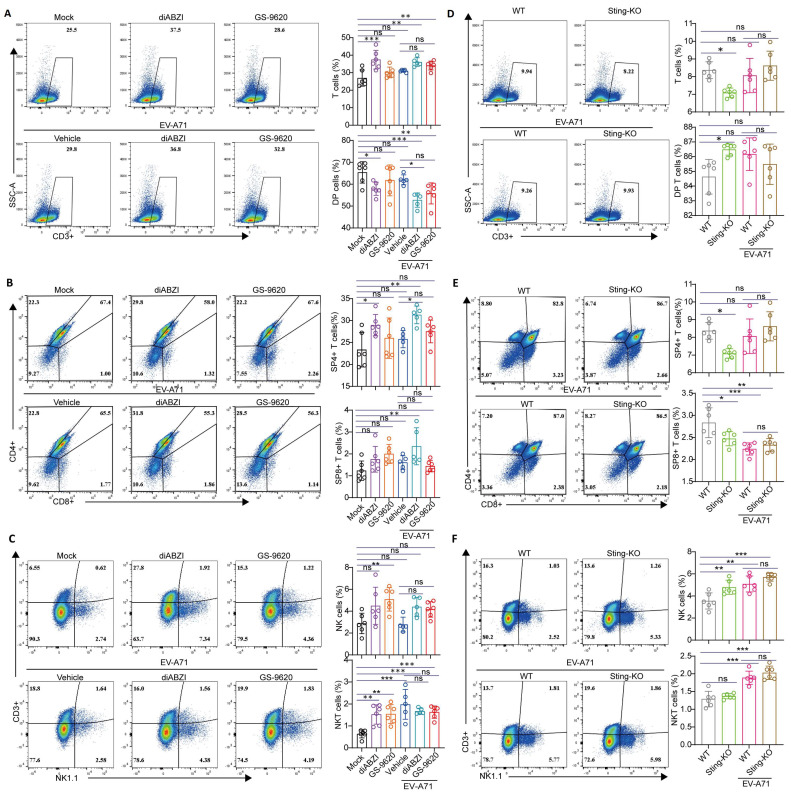
Impact of STING on immune cells in the thymus. (**A**–**C**) Ten-day-old C57BL/6J mice were intraperitoneally inoculated with 10 LD_50_ of EV-A71-H-MA. One hour after virus infection, the mice were treated with diABZI or GS-9620 once daily for 3 days. Flow cytometry was used to measure the percentage of different cell types in the thymus (*n* = 5 or 6). (**D**–**F**) Nine-day-old WT mice and Sting-KO mice were intraperitoneally inoculated with 1 LD_50_ of EV-A71-H-MA. Flow cytometry was used to measure the percentage of different cell types in the thymus (*n* = 6). Data are from one experiment with *n* = 5 or 6 mice per group. The color gradient from blue to red indicates a progressive increase in cell density. Wireframe represents flow cytometry gating. * *p* < 0.05, ** *p* < 0.01, *** *p* < 0.001, one-way ANOVA with Holm–Sidak multiple comparisons test or two-tailed Student’s *t*-test, and “ns” indicates no significant difference (A–F).

The thymus, as an important lymphatic organ, serves as the primary site of differentiation, development, and maturation of T lymphocytes [33]. T cells undergo several stages in the thymus from progenitor cells to mature T cells, from CD4−CD8− double negative (DN) cells to CD4+CD8+ double positive (DP) cells, and finally to CD4+ or CD8+ single positive (SP) cells [34]. To further elucidate the impact of STING activation on the immune response, flow cytometry was employed to analyze immune cells isolated from the thymus. The results demonstrated that upon treatment with diABZI, the number and frequency of T thymocytes increased, whereas the percentage of DP thymocytes decreased (Figure 7A and Supplementary Figure S5A). Concurrently, the numbers and percentages of SP4+ and SP8+ thymocytes rose (Figure 7B and Supplementary Figure S5B), which is consistent with previous study revealing that activated STING in thymic stroma increases single positive thymocytes [35]. After GS-9620 treatment, although the increase in the proportions of T, SP4+, and SP8+ thymocytes did not reach statistical significance, the upregulation of their cell numbers was statistically significant (Figure 7A,B and Supplementary Figure S5A,B). In addition, both diABZI and GS-9620 treatments significantly augmented the number and frequency of NKT thymocytes (Figure 7C and Supplementary Figure S5C). In contrast, the frequencies of T, SP4+, and SP8+ thymocytes were strikingly decreased in the absence of STING, whereas the number and percentage of DP thymocytes was significantly enhanced (Figure 7D,E and Supplementary Figure S5D,E), which displayed a trend completely opposite that of STING activation. Moreover, STING knockout exerted no impact on the number and percentage of NKT thymocytes, but slightly upregulated the number and percentage of NK thymocytes (Figure 7F and Supplementary Figure S5F).

In addition, the sexual maturity of mice typically occurs at approximately 5 weeks of age, but the physiological rapid growth phase persists until they reach three months old. Given that the mice utilized in the EV-A71 infection model were about 2 weeks old, we further studied the impact of STING activation on T-cell development in the thymus at various weeks before and after sexual maturity. Analogous to the findings in 2-week-old mice, the results demonstrated that following treatment with diABZI, regardless of the mice’s age in different weeks, the number and frequency of T thymocytes increased, the number and frequency of DP thymocytes decreased, and the numbers and frequencies of SP4+ and SP8+ thymocytes were enhanced (Supplementary Figure S6). In summary, our results strongly indicate that STING activation promotes the transformation of T cells from DP to SP and affects the development of T cells in the thymus.

## 3. Discussion

Accumulating evidence suggests that the STING pathway can enhance immune system activity, aiding cells in resisting various pathogens and even tumorigenesis [36,37]. As a pivotal signaling molecule in the innate immune system, STING can activate downstream interferon signaling pathways by recognizing viral DNA or dsRNA, thereby inducing antiviral immune responses. This study systematically investigated the immunomodulatory role of STING in EV-A71 infection and its potential antiviral mechanisms through in vivo experiments. Our findings demonstrate that the STING-specific agonist diABZI significantly improved the survival rate of EV-A71-infected mice and alleviates clinical symptoms (Figure 1). DiABZI treatment markedly reduced viral titers and VP1 protein expression in the hind-limb muscles of EV-A71-infected mice (Figure 2). Conversely, STING knockout significantly enhanced EV-A71 replication and infection severity (Figure 1). STING knockout mice exhibited higher viral titers and more severe clinical symptoms post-infection (Figure 2), underscoring the critical role of STING in limiting EV-A71 infection.

Our research also revealed that STING influences EV-A71 replication by modulating immune and inflammatory responses. DiABZI treatment significantly stimulated the phosphorylation of STING, TBK1, IRF3, and STAT1 in mouse muscle tissues, upregulating the expression of interferon-stimulated genes (ISGs) without affecting LC3 conversion (LC3-I to LC3-II) (Figure 3). These results indicate that STING activation suppresses EV-A71 replication by enhancing the host’s antiviral immune response rather than through autophagy regulation. Interestingly, GS-9620, a TLR7 agonist, also stimulated the phosphorylation of STING and downstream proteins in mouse muscle tissues (Figure 3), suggesting a potential cross-regulation between the STING and TLR signaling pathways. Additionally, although STING knockout did not affect the phosphorylation of P65, TBK1, and IRF3 stimulated by EV-A71 infection (Figure 3C), it did downregulate the mRNA level of Isg15 (Figure 3E), indicating that other PRR signaling pathways can partially compensate for the impact of STING knockout on the IFN signaling pathway. In addition, perhaps because our sampling time point was on the 4 dpi, we did not detect a significant stimulation of STING phosphorylation by EV-A71 infection. Instead, EV-A71 infection inhibited STING phosphorylation to a certain extent. This is consistent with the report by Zheng et al., that is, EV-A71 infection triggers STING activation in a cGAS-dependent manner, but EV-A71 2Apro in the later stage of infection can inhibit the activity of cGAS-STING [15]. Furthermore, diABZI treatment significantly reduced the levels of pro-inflammatory cytokines/chemokines in the serum (Figure 4A). Pro-inflammatory factors such as IL-6 can initiate B lymphocyte responses and acute-phase reactions, aiding the body in resisting viral infections [38]. However, excessive responses can lead to systemic inflammatory reactions (i.e., “cytokine storm”), exerting negative regulatory effects [39]. STING plays a crucial role in regulating the delicate balance of the immune response, and overactivation of STING has been associated with several autoinflammatory and autoimmune diseases, including STING-associated infantile vascular disease (SAVI) and inflammatory bowel disease (IBD) and systemic lupus erythematosus [40,41,42]. However, moderate STING activation is essential to ward off viral infection. Precise and strict regulation of the activity of the cGAS-STING signaling pathway, especially STING, is very important for maintaining immune homeostasis. STING activation reduces the levels of pro-inflammatory cytokines/chemokines in the serum, potentially preventing cytokine storms, suggesting that STING activation can mitigate tissue inflammatory damage caused by EV-A71 infection by modulating inflammatory responses. Of course, if STING agonists can be used in the treatment of infectious diseases or tumors in the future, we still need to pay attention to factors such as the dosage and treatment cycle of STING agonists to prevent side effects caused by the excessive activation and disruption of immune homeostasis.

To further elucidate the immunomodulatory effects of STING activation, we examined its impact on immune cells in the thymus and spleen. Although T cells, NK cells, macrophages, and neutrophils have different functions, they share similar characteristics in producing various inflammatory factors and chemokines, recruiting more cells of the same type, and exhibiting synergistic effects in antiviral or anti-inflammatory processes. Our results show that diABZI treatment increased the total percentages of thymic T cells, NK cells, NKT cells, and SP cells (Figure 7), while STING knockout reduced the total proportion of T thymocytes (Figure 7). DiABZI treatment significantly increased the total frequencies of T cells, activated CD4+ T cells, activated CD8+ T cells, NKT cells, various types of NK cells, neutrophils, macrophages, and DC cells in the spleen (Figure 5, Figure 6 and Figure 7). However, STING knockout impeded thymic T cell development and significantly reduced thymic T cell frequencies. While splenic immune cell proportions remained unchanged, the activation status and secretory capacities of CD8+ T cells and NK cells were impaired during EV-A71 challenge. Studies indicate that the cGAS-STING signaling pathway regulates the activation and maturation of DCs, and STING knockout significantly hinders DC activation and suppresses the activation of γδ T cells, Th1, and Th17 cells [43]. Collectively, these findings indicate that STING is critical for immune cell development and effector function.

STING plays a key role not only in antiviral immunity but also influences the establishment of adaptive immunity by regulating thymic T cell development. Notably, STING exhibits dynamic expression and strict regulation during thymic T cell development. Research shows that STING is highly expressed in DN and SP thymocytes but is temporarily silenced during the DP stage through epigenetic mechanisms [44]. This expression pattern has significant physiological importance. Silencing STING at the DP stage helps avoid STING-mediated cell death, ensuring T cells successfully undergo positive and negative selection. In the context of EV-A71 infection, STING activation (e.g., via diABZI treatment) promoted the transition from DP to SP stages, manifested by an increased proportion of SP4+ and SP8+ cells and a decreased proportion of DP cells. This aligns with the findings reported by Deng et al. that STING activation in thymic epithelial cells promotes SP cell expansion [36]. This accelerated or biased T cell development within the thymus might facilitate the rapid output of effector T cells during the early stages of viral infection, thereby enhancing the antiviral immune response. We believe that STING plays a key role in both thymic T cell development and anti-EV-A71 immunity. In the thymus, it regulates the transition of T cell development stages through spatiotemporal specific expression. During the infection process, it inhibits viral replication and regulates inflammatory responses through type I interferon signaling. There is a delicate balance between the autoimmune predisposition caused by STING activation and the protective effect of STING in viral infections. Future research should further explore the interaction between STING’s regulation of T cell lineages within the thymus and antiviral immunity, as well as its potential double-edged sword effect in immunotherapy.

Increasing research indicates that the STING pathway, whether induced or inhibited, is a promising therapeutic target in cancer, antiviral, anti-inflammatory responses, and vaccine development [45,46]. This study highlights the critical role of STING in combating EV-A71 infection and elucidates its mechanism of suppressing viral replication by modulating immune and inflammatory responses. STING activation not only directly inhibits EV-A71 replication but also enhances the host’s immune response to mitigate infection-induced tissue damage. These findings provide new insights for developing immunotherapeutic strategies against EV-A71 infection and lay the groundwork for further research on STING’s role in antiviral immunity. Future studies should further explore the universal role of STING in different viral infections and how to optimize antiviral immune responses through the precise regulation of STING activity.

## 4. Materials and Methods

### 4.1. Cells and Viruses

Vero cells were acquired from the American Type Culture Collection (ATCC) and maintained in modified Eagle’s medium (Invitrogen) containing 10% heat-inactivated fetal bovine serum (FBS, Gibco, Grand Island, NY, USA) along with penicillin (100 U/mL) and streptomycin (100 μg/mL, Invitrogen) under standard culture conditions (37 °C, 5% CO_2_).

The EV-A71 strain H was sourced from ATCC, while the mouse-adapted variant (EV-A71-H-MA) employed for the in vivo studies was generated in-house through serial passage in mice. All viral stocks were propagated in Vero cells [47,48,49].

### 4.2. Mice

C57BL/6J mice (SiPeiFu Biotechnology Co., Ltd., Beijing, China) and Sting-deficient (Sting-KO) mice (Shanghai Model Organisms Center Inc., Shanghai, China) were housed in a controlled SPF environment under a 12 h light/dark cycle with free access to food and water.

### 4.3. Compounds

Diamidobenzimidazole (diABZI) (cat no. S8796) was purchased from Beijing Ouhe Technology Co., Ltd. (Beijing, China) GS-9620 was purchased from MedChemExpress (Monmouth Junction, NJ, USA). For the in vivo experiments, diABZI or GS-9620 was dissolved in saline (0.9% NaCl) with 1% DMSO (Sigma, St. Louis, MO, USA) and 40% PEG400 (Aladdin, Shanghai, China).

### 4.4. In Vivo Antiviral Study of diABZI and GS-9620

The antiviral efficacy of diABZI and GS-9620 was assessed in a murine model of EV-A71-H-MA infection. All procedures involving mice were conducted under animal biosafety level 2 conditions and in accordance with protocols approved by the Animal Care and Welfare Committee of the Institute of Medicinal Biotechnology, Chinese Academy of Medical Sciences & Peking Union Medical College.

To evaluate mortality and morbidity, 10-day-old C57BL/6J pups (No gender distinction) were intraperitoneally inoculated with a lethal dose (10 LD_50_) of EV-A71-H-MA. Commencing 1 h post-infection, mice received daily intraperitoneal administration of either diABZI (0.5 mg/kg or 0.1 mg/kg) or GS-9620 (5 mg/kg) for three consecutive days. The Mock group, which served as an uninfected control, received the solvent only. The Vehicle group, which served as an infection control, was infected and administered the solvent. Over a period of 14 days, the survival of the animals and the clinical signs of disease were monitored daily using a double-blind method. Clinical scoring was performed based on established criteria: 0 (healthy), 1 (ruffled fur), 2 (limb weakness), 3 (hind limb paralysis), and 4 (dead) [22,47,48,49].

For analysis of viral replication and tissue pathology, a parallel experiment was performed using the same infection and treatment regimen. Mice were euthanized at 3 days post-infection (dpi). Hind-limb muscle tissues were collected for quantification of viral load (viral titer), viral RNA, and host protein expression. Sections of the spleen and thymus were processed for flow cytometric analysis. Additional tissue samples were fixed in 10% neutral buffered formalin for subsequent histological examination by hematoxylin and eosin (H&E) staining and immunofluorescence (IF) assay. Serum samples were isolated for cytokine and chemokine profiling [47,48,49].

### 4.5. Effect of STING Knockout on EV-A71 Infection In Vivo

The critical role of the STING signaling pathway in host defense against EV-A71 was investigated using *Sting*-knockout (KO) mice. Nine-day-old wild-type (WT) and *Sting*-KO C57BL/6J mice were challenged intraperitoneally with a sublethal dose (1 LD_50_) of EV-A71-H-MA. Survival rates and clinical symptoms were recorded daily for two weeks, with clinical scores assigned as described above.

To examine viral propagation and histopathological changes, infected WT and *Sting*-KO mice were dissected at 3 dpi. The detection of relevant indicators was the same as that of the STING activation experiment.

### 4.6. Quantitative Reverse-Transcription Polymerase Chain Reaction (qRT-PCR) Assay

Muscle tissue total RNA was extracted with TRIzol reagent and a magnetic bead-based RNA extraction kit (both from Genfine Biotech, Changzhou, China), adhering to the provided protocols. Quantitative reverse transcription PCR (qRT-PCR) was then conducted on an ABI 7500 Fast Real-Time PCR system (Applied Biosystems, Carlsbad, CA, USA) employing the TransScript II Green One-Step qRT-PCR SuperMix (TransGen Biotech, Beijing, China). Relative ratios were determined based on the 2-∆∆CT method. The sequences of all primers utilized are listed in Supplementary Table S1 [47,48,49].

### 4.7. Western Blot (WB) Assay

Total cellular proteins were lysed with T-PER™ tissue protein extraction reagent (Thermo Fisher Scientific, Waltham, MA, USA) supplemented with a Halt protease and phosphatase inhibitor cocktail (Thermo). Western blot was then carried out following standard procedures, with specific antibody information provided in Supplementary Table S2 [47,48,49].

### 4.8. Virus Titer Determination in the Infected Mice

Virus titers were determined by a standard CPE-based assay in Vero cells. Muscle homogenates, prepared in cold MEM using a Precellys Evolution homogenizer (Bertin, France), were centrifuged to collect the supernatant. This supernatant was then used to infect pre-seeded Vero cells (3 × 10^4^ cells/well in 96-well plates). After a 1 h adsorption in serum-free medium at 37 °C, the cells were maintained in MEM with 2% FBS for 72 h. The TCID_50_ value was finally calculated using the Reed and Muench method [48,50].

### 4.9. Immunofluorescent (IF) Staining

For IF staining, formalin-fixed and paraffin-embedded tissues were sectioned at a thickness of 3 μm. The sections were deparaffinized with xylene and rehydrated in ethanol, then placed into 3% hydrogen peroxide to block endogenous peroxidase activity. Subsequently, the tissue sections were incubated with primary antibodies at 4 °C overnight and then incubated with species-specific secondary antibodies at room temperature (RT). The antibodies used are shown in Supplementary Table S2. Images were acquired using a 3D Histech MIDI panoramic scanner (3D Histech, Budapest, Hungary).

### 4.10. Multi-Cytokine/Chemokine Measurement by Luminex-Based Cytokine Bead Array

All serum from mice were detected for cytokine and chemokine expression by using Bio-Plex Pro^TM^ Assays (Bio-Rad) according to the manufacturer’s recommendations. Plates were read by a Bio-Plex Suspension Array system (Bio-Rad). Data were collected and analyzed by Bio-Plex Manager software. A five-parameter regression formula was used to calculate sample concentration from the standard curves [51].

### 4.11. Flow Cytometry

Thymocytes and splenocytes were obtained by gently grinding these organs. After filtration and centrifugation, the single cell suspensions were treated with 1 × RBC (red blood cell) Lysing Buffer (BD, 555899) to remove the erythrocytes. Prior to cell staining, cell viability was determined using the trypan blue assay. The survival rate of splenocytes was 84% to 90%, and that of thymocytes was 86% to 91%. For cell analysis, cells were resuspended in MACS buffer (PBS with 0.5% FBS and 2 μM EDTA), blocked with FcR mouse antibody (biolegend, 101320) and stained with fluorescently labelled specific antibodies (Supplementary Table S2). For the analysis of Granzyme B, IFN-γ, and TNF-α, cells were stimulated with PMA (Solarbio), Ionomycin (sigma), brefeldin A (Solarbio), and GolgiStop protein transport inhibitor (BD) at 37 °C for 4 h. Prior to intracellular staining, cells were first subjected to surface marker staining. Subsequently, the stained cells were fixed with fixation buffer (BD, 51-2090KZ, Frank Lakes, NJ, USA) and incubated overnight at 4 °C. The samples were acquired by Beckman Coulter CytoFLEX (Brea, CA, USA), and the acquired data were analyzed with FlowJo 10.8.1 software. The Flow cytometry gating strategy is shown in Supplementary Figure S7.

### 4.12. Quantification and Statistical Analysis

GraphPad Prism 9.0 and SPSS 13.0 software were employed for statistical analyses. Quantitative data are expressed as the mean ± SD and were analyzed by Student’s *t*-test or one-way ANOVA. The Log-Rank (Mantel-Cox) test was used for survival analysis, while the Ridit assay was applied for clinical score evaluation. Statistical significance was set at *p* < 0.05.

## Data Availability

The data that support the findings of this study are available on request from the corresponding author. The data are not publicly available due to privacy or ethical restrictions.

## Supplementary Materials

The following supporting information can be downloaded at: https://www.mdpi.com/article/10.3390/ijms262311441/s1.
