# Correction: Wang et al. STING Restricts EV-A71 Infection by Regulating T Cell Development and Enhancing Immune Cell Effector Function. *Int. J. Mol. Sci.* 2025, *26*, 11441

**DOI:** 10.3390/ijms27135853

**Published:** 2026-06-29

**Authors:** Huiqiang Wang, Ya Wang, Shuo Wu, Lijun Qiao, Wen Sheng, Haiyan Yan, Kun Wang, Ge Yang, Jiandong Jiang, Yuhuan Li

**Affiliations:** 1 CAMS Key Laboratory of Antiviral Drug Research, Beijing Key Laboratory of Technology and Application for Anti-Infective New Drugs Research and Development, NHC Key Laboratory of Biotechnology of Antibiotics, Institute of Medicinal Biotechnology, Chinese Academy of Medical Sciences and Peking Union Medical College, Beijing 100050, China; wangya@imb.pumc.edu.cn (Y.W.); wushuo@imb.pumc.edu.cn (S.W.); qiaolijun@imb.pumc.edu.cn (L.Q.); shengwen@imb.pumc.edu.cn (W.S.); yanhaiyan@imb.pumc.edu.cn (H.Y.); wangkun@imb.pumc.edu.cn (K.W.); yangge@imb.pumc.edu.cn (G.Y.); jiangjiandong@imb.pumc.edu.cn (J.J.); 2 Division for Medicinal Microorganism-Related Strains, CAMS Collection Center of Pathogenic Microorganisms, Chinese Academy of Medical Sciences and Peking Union Medical College, Beijing 100050, China; 3 State Key Laboratory of Bioactive Substances and Functions of Natural Medicines, Institute of Medicinal Biotechnology, Chinese Academy of Medical Sciences and Peking Union Medical College, Beijing 100050, China

In the original publication [1], reference [27] has been retracted and therefore requires revision.

The previous reference:

27. Shang, W.; Qian, S.; Fang, L.; Han, Y.; Zheng, C. Association study of inflammatory cytokine and chemokine expression in hand foot and mouth disease. *Oncotarget* **2017**, *8*, 79425–79432.

has been replaced with:

27. Shao, P.; Wu, X.; Li, H.; Wu, Z.; Yang, Z.; Yao, H. Clinical significance of inflammatory cytokine and chemokine expression in hand, foot and mouth disease. *Mol. Med. Rep.* **2017**, *15*, 2859–2866.

The authors confirm that the scientific conclusions of the original paper remain unchanged. This correction has been approved by the Academic Editor and has been updated in the published version.
