# Supplementary material for: STING Restricts EV-A71 Infection by Regulating T Cell Development and Enhancing Immune Cell Effector Function
Source: Int J Mol Sci. 2025 Nov 26;26(23):11441. doi: 10.3390/ijms262311441 (PMC12692614; doi:10.3390/ijms262311441)
Supplement: Supplementary file 1 [file ijms-26-11441-s001.zip › Supplementary material.pdf]

Supplementary Materials for

STING restricts EV-A71 infection by regulating T cell development and enhancing immune cell effector function

Huiqiang Wang [a,b #*], Ya Wang [a,b #], Shuo Wu [a,b,c], Lijun Qiao [a,b], Wen Sheng [a,b], Haiyan Yan [a,b], Kun Wang [a,b], Ge Yang [a,b], Jiandong Jiang [a,c] and Yuhuan Li [a,b,c *]

Correspondence to: Huiqiang Wang (wanghuiqiang@imb.pumc.edu.cn) or Yuhuan Li (liyuhuan@imb.pumc.edu.cn).

This PDF file includes:
Figures. S1 to S7
Tables S1 to S2

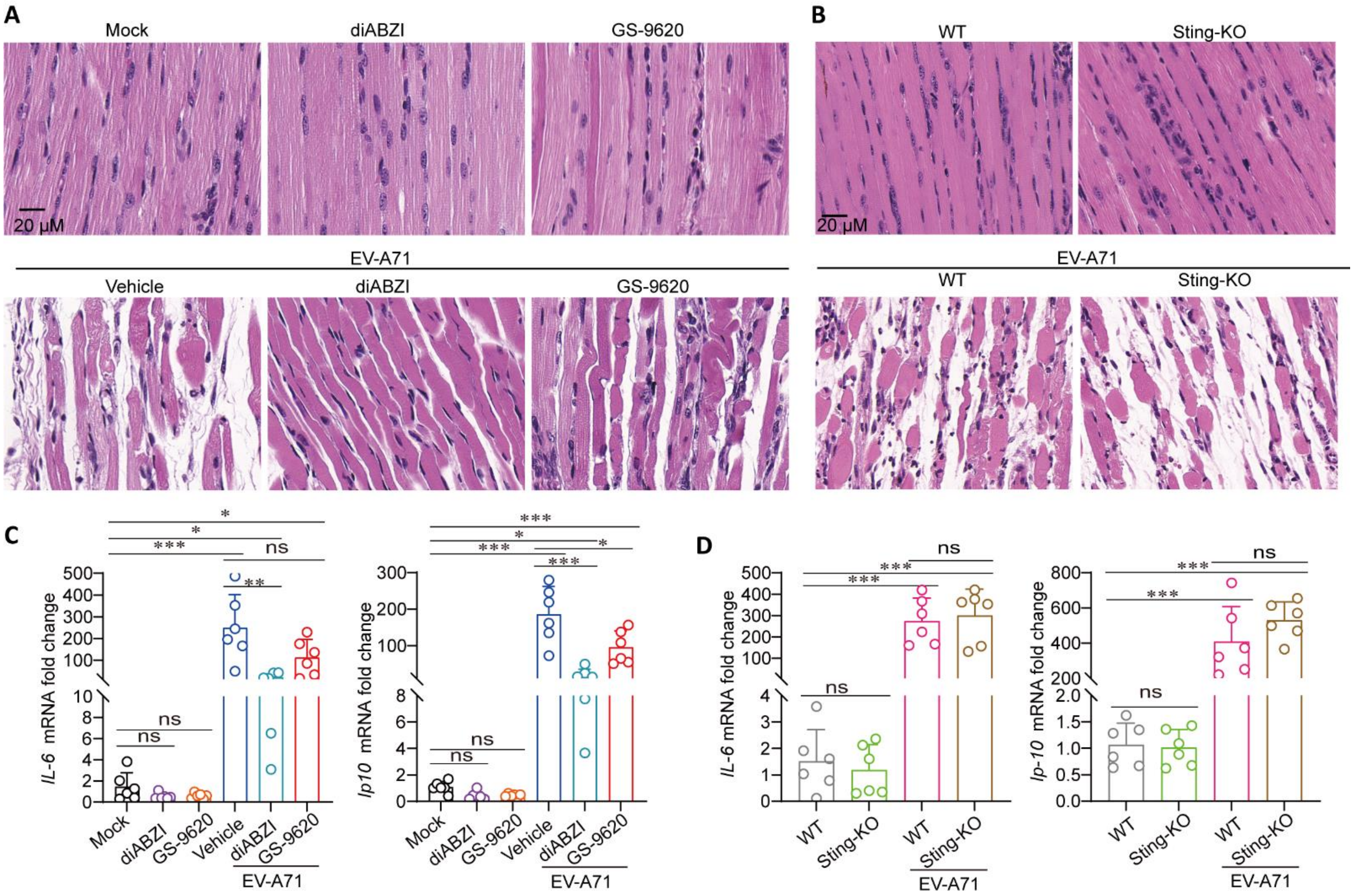

Figure S1. The influence of STING regulation on EV-A71 infection-induced muscle inflammatory injury. (A,B) Paraffin-embedded sections of muscle tissues were prepared from mice and examined with H&E stain. (C,D) Muscles were detected by qRT-PCR assay (*n* = 6). Data are from one experiment with *n* = 6 mice per group. *$P<0.05$, one-way ANOVA with Holm-Sidak multiple comparisons test or two-tailed Student's t-test.

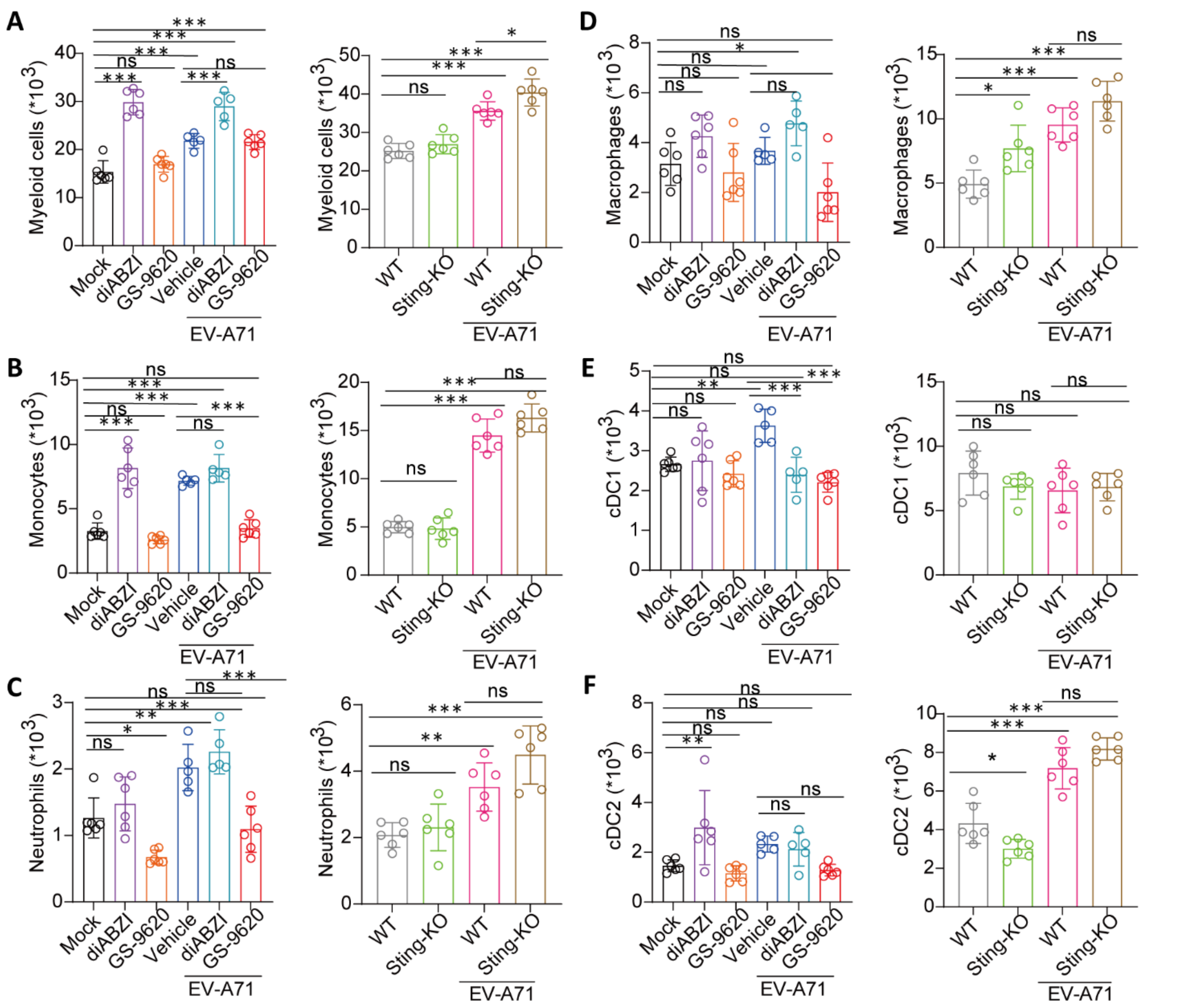

Figure S2. Modulation of splenic myeloid cell populations by STING. The numbers of total myeloid cells (A), monocytes (B), neutrophils (C), macrophages (D), and distinct dendritic cell (DC) subsets (E-F) were analyzed via flow cytometry (*n* = 5 or 6). Data are from one experiment with *n* = 5 or 6 mice per group. *$P<0.05$, one-way ANOVA with Holm-Sidak multiple comparisons test or two-tailed Student's t-test (A-F).

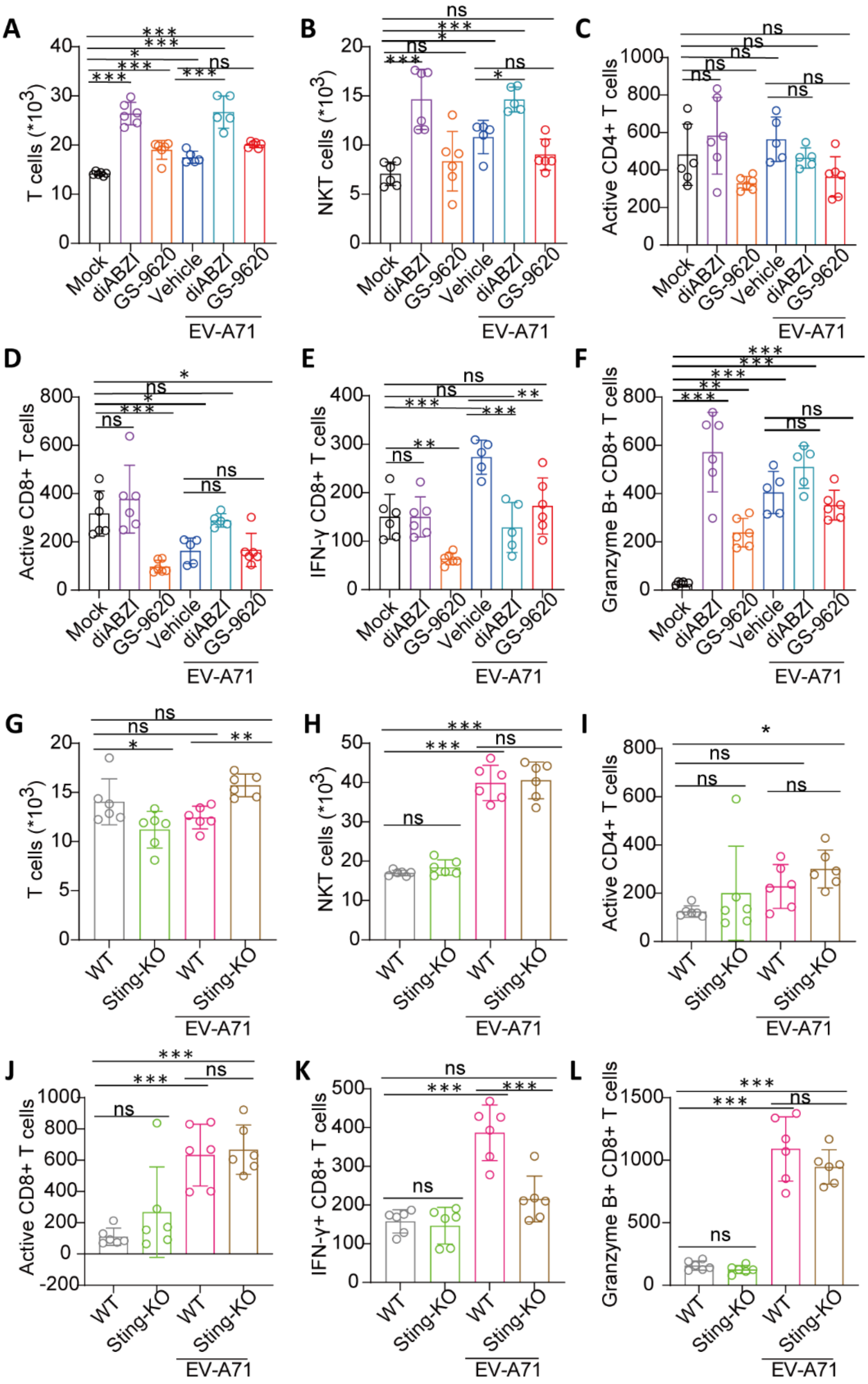

Figure S3. Effect of STING on splenic T cells. (A-F) 10-day-old C57BL/6J mice were intraperitoneally inoculated with 10 LD50 of EV-A71-H-MA. One hour after virus infection, the mice were treated with diABZI or GS-9620 once daily for 3 days. The number of different types of T cells in spleen was measured by flow cytometry (*n*= 5 or 6). (G-L) 9-day-old C57BL/6J WT mice and Sting-KO mice were intraperitoneally inoculated with 1 LD50 of EV-A71-H-MA. The number of different types of T cells in spleen was measured by flow cytometry (*n* = 6). Data are from one experiment with *n* = 5 or 6 mice per group. *P<0.05, one-way ANOVA with Holm-Sidak multiple comparisons test or two-tailed Student's t-test (A-L).

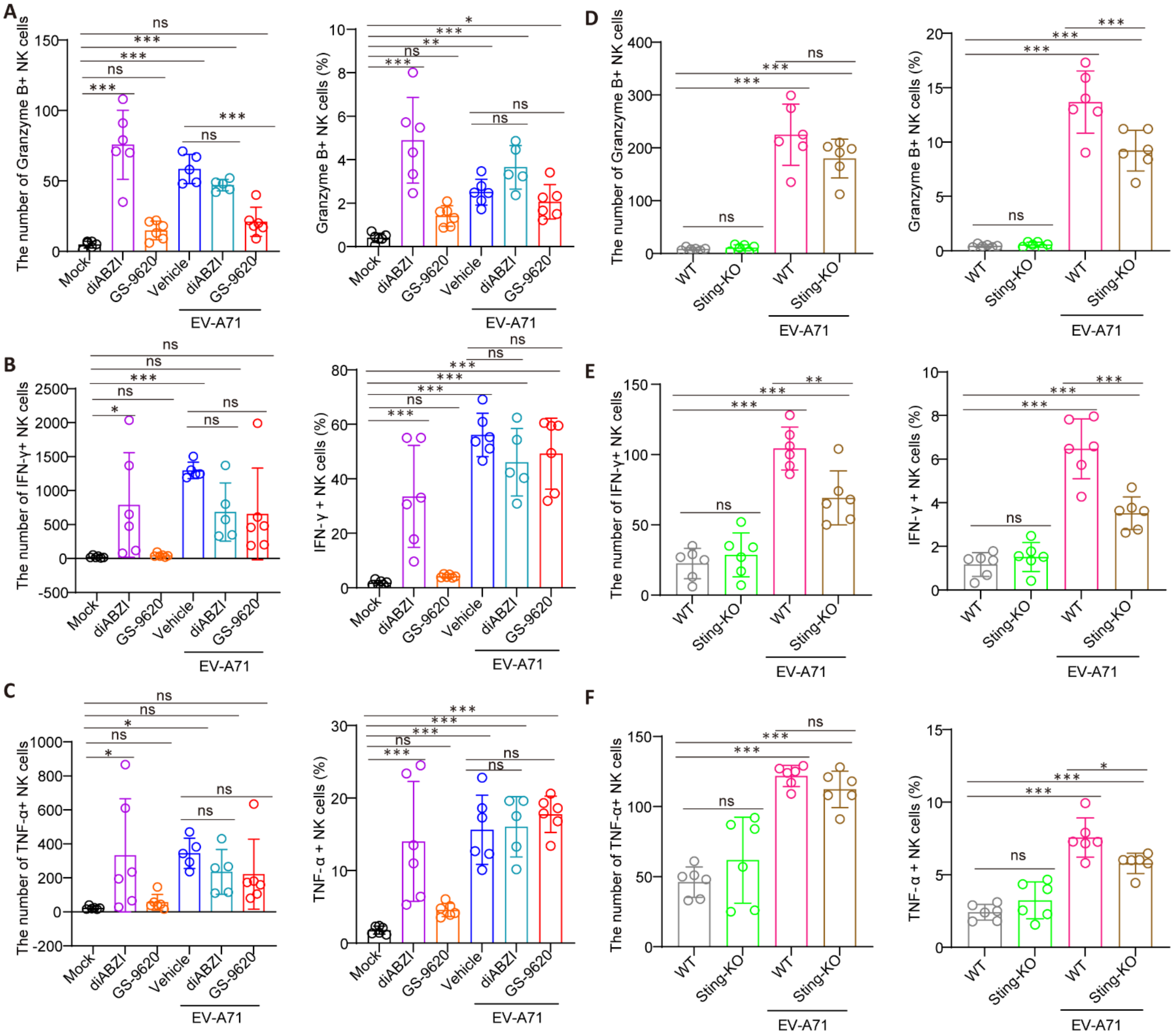

Figure S4. The Impact of STING on the functional properties of splenic NK cells. (A-C) 10-day-old C57BL/6J mice were intraperitoneally inoculated with 10 LD_{50} of EV-A71-H-MA. One hour after virus infection, the mice were treated with diABZI or GS-9620 once daily for 3 days. The number and percentage of Granzyme B+ NK cells, IFN-γ+ NK cells and TNF-α+NK cells in spleen were measured by flow cytometry (*n* = 5 or 6). (D-F) 9-day-old WT mice and Sting-KO mice were intraperitoneally inoculated with 1 LD_{50} of EV-A71-H-MA. The number and percentage of Granzyme B+ NK cells, IFN-γ+ NK cells and TNF-α+NK cells in spleen were measured by flow cytometry (*n* = 6). Data are from one experiment with *n* = 5 or 6 mice per group. *$P<0.05$*, one-way ANOVA with Holm-Sidak multiple comparisons test or two-tailed Student's t-test (A-F).

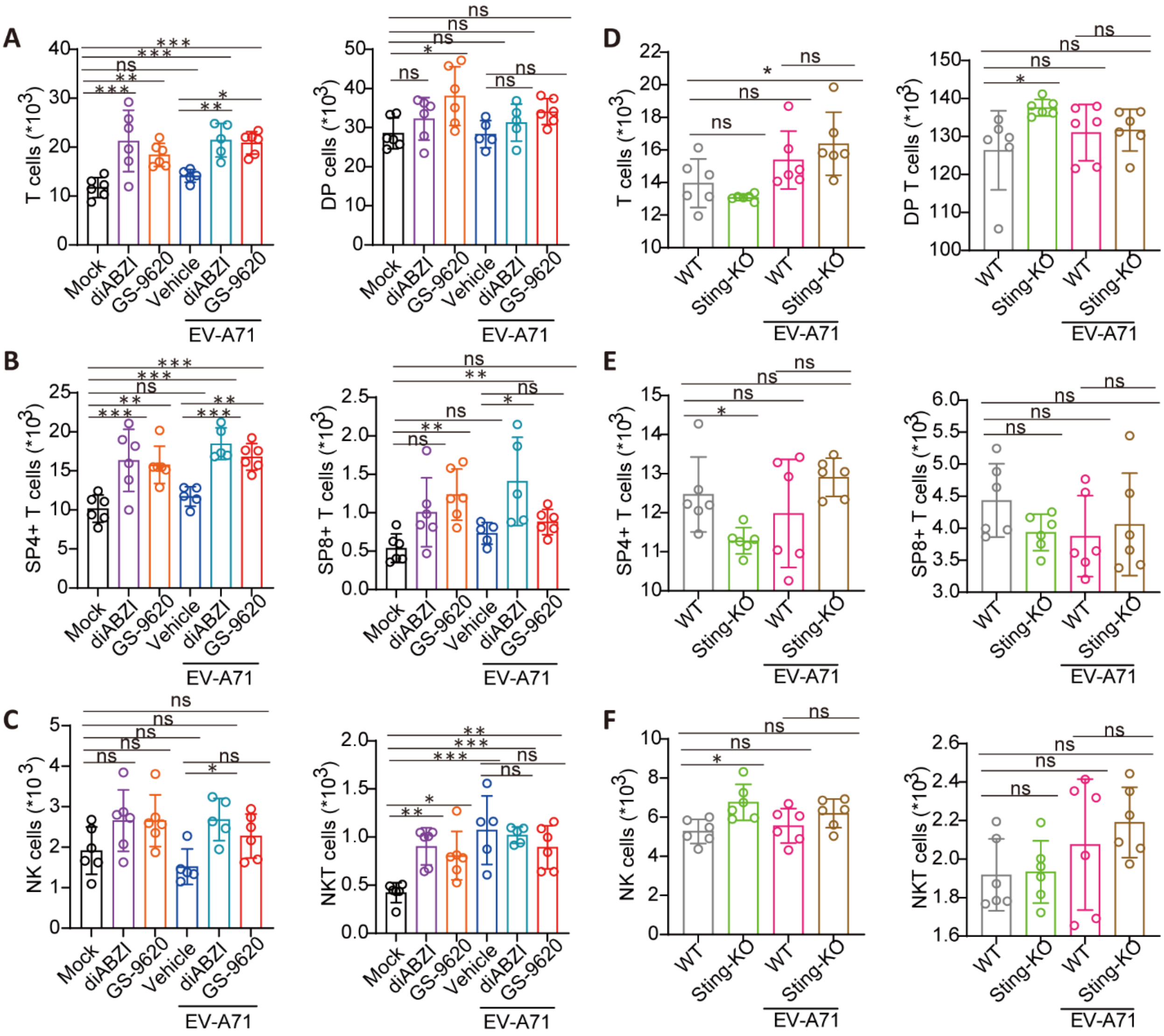

Figure S5. Impact of STING on immune cells in the thymus. (A-C) 10-day-old C57BL/6J mice were intraperitoneally inoculated with 10 LD_{50} of EV-A71-H-MA. One hour after virus infection, the mice were treated with diABZI or GS-9620 once daily for 3 days. Flow cytometry was used to measure the number of different cell types in the thymus (n = 5 or 6). (D-F) 9-day-old WT mice and Sting-KO mice were intraperitoneally inoculated with 1 LD_{50} of EV-A71-H-MA. Flow cytometry was used to measure the number of different cell types in the thymus (n = 6). Data are from one experiment with n = 5 or 6 mice per group. *P<0.05, one-way ANOVA with Holm-Sidak multiple comparisons test or two-tailed Student's t-test (A-F).

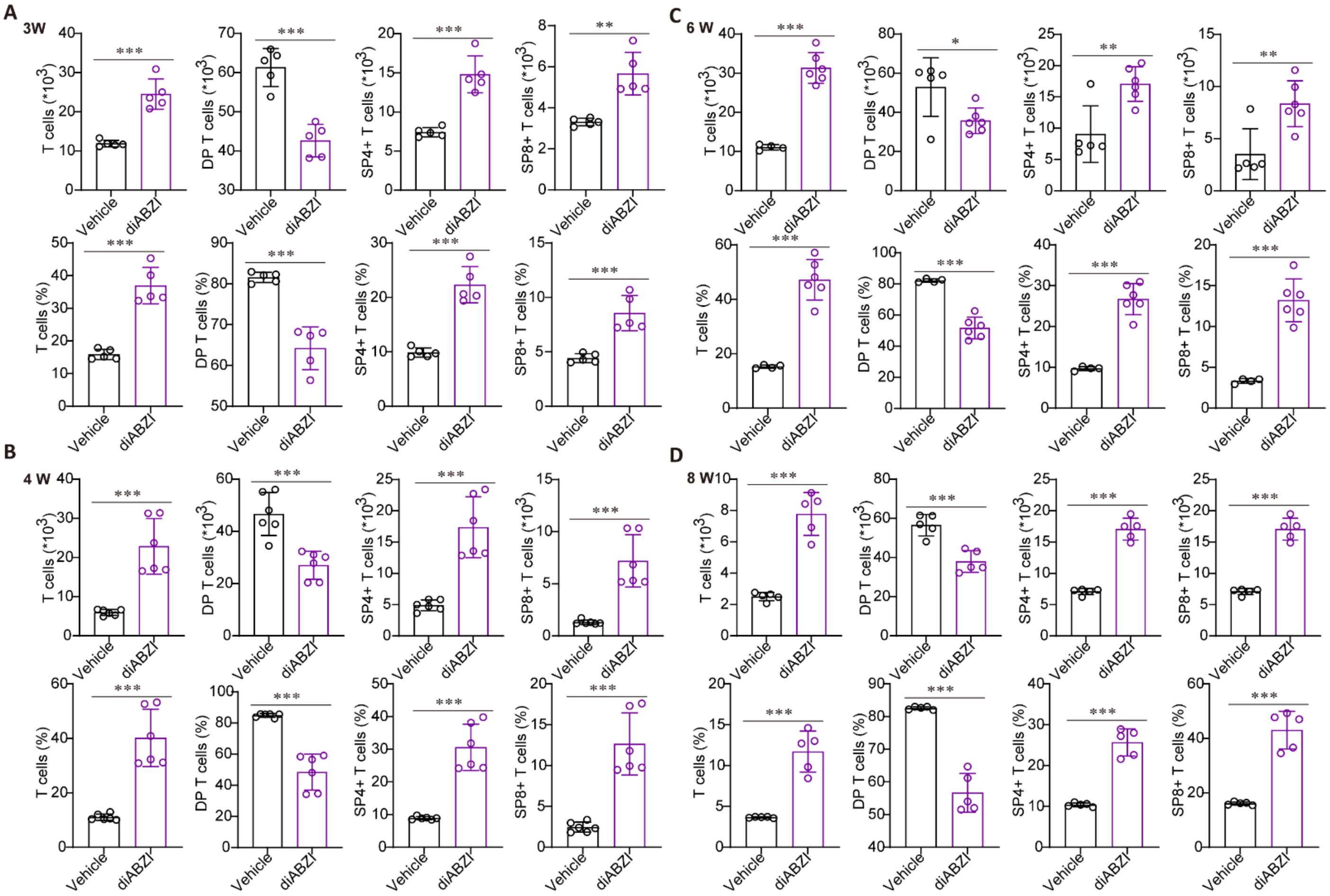

Figure S6. Impact of STING activation on T-cell development in the thymus of mice at different weeks of age. (A-D) C57BL/6 mice of 3, 4, 6 or 8 weeks of age were treated with diABZI once daily for 3 days. Flow cytometry was used to measure the number and percentage of different cell types in the thymus (*n* = 5 or 6). Data are from one experiment with *n* = 5 or 6 mice per group. $^{*}P<0.05$, two-tailed Student's t-test (A-D).

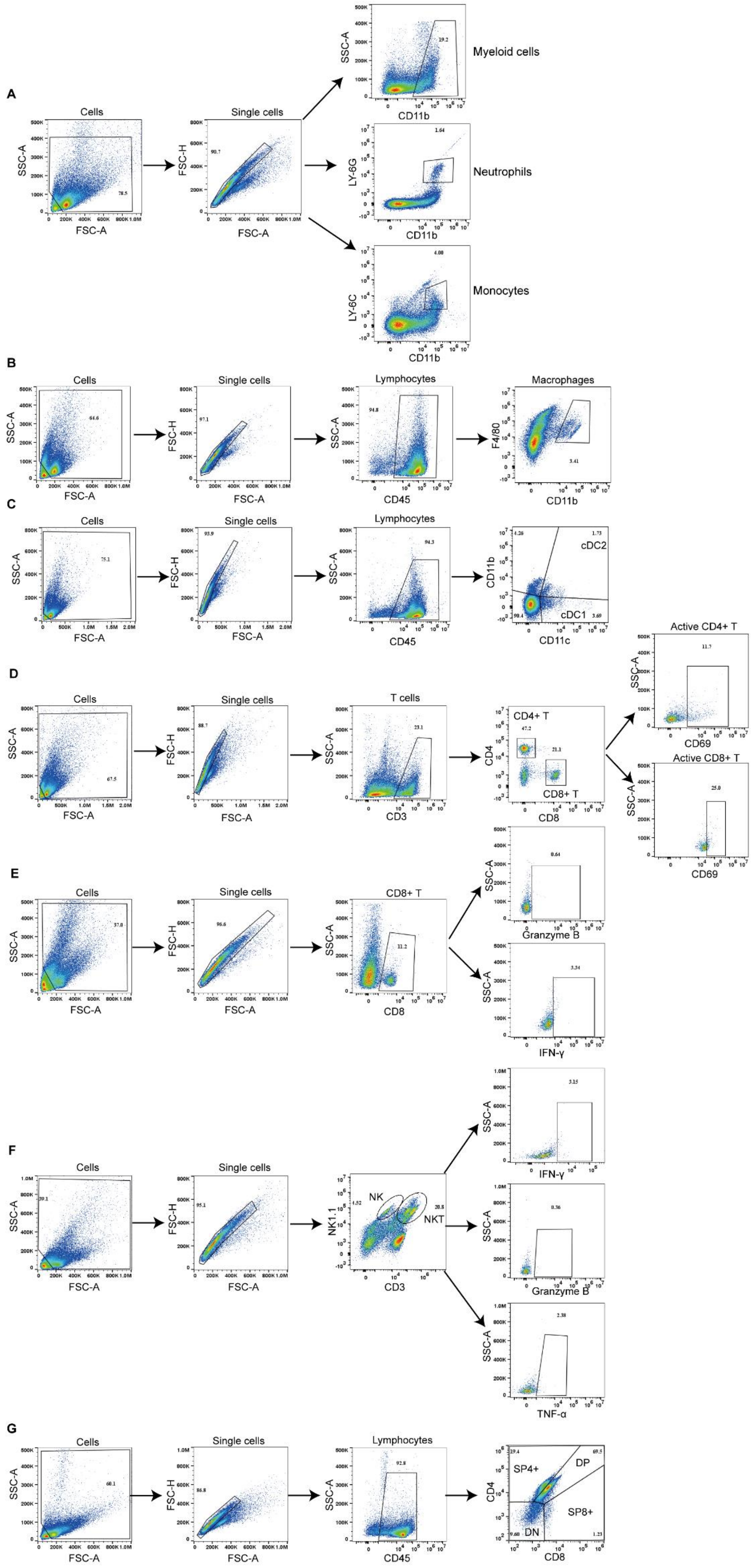
A
Cells
Single cells
Myeloid cells
Neutrophils
Monocytes
SSC-A
FSC-A
FSC-H
CD11b
LY-6G
LY-6C
B
Lymphocytes
Macrophages
CD45
F4/80
C
CD11c
cDC2
cDC1
D
T cells
CD3
CD4
CD8
CD4+ T
CD8+ T
Active CD4+ T
Active CD8+ T
CD69
E
CD8+ T
Granzyme B
IFN-γ
F
NK
NKT
NK1.1
TNF-α
G
SP4+
DP
SP8+
DN

Figure S7. Flow cytometry gating strategy. (A-F) The gating strategy of myeloid cells (A), neutrophils (A), monocytes (A), macrophages (B), cDC1 (C), cDC2 (C), T cells (D), CD4+ T cells (D), CD8+ T cells (D), active CD4+ T cells (D), active CD8+ T cells (D), Granzyme B+ CD8+ T cells (E), IFN-γ+ CD8+ T cells (E), IFN-γ+ NK cells (F), Granzyme B+ NK cells (F), TNF-α+ NK cells (F) in the spleen or thymus from mice. (G) The gating strategy of DN, DP, SP4+, SP8+ T cells in the thymus from mice.

Table S1. qRT-PCR primer sequences.

Primer name	Primer Sequence
Ifnβ F	AGCTCCAAGAAAGGACGAACAT
Ifnβ R	GCCCTGTAGGTGAGGTTGATCT
Il-6 F	AACGATGATGCACTTGCAGA
Il-6 R	GAGCATTGGAAATTGGGGTA
Ifit1 F	CAGAAGCACACATTGAAGAA
Ifit1 R	TGTAAGTAGCCAGAGGAAGG
Isg15 F	CTGTACCACTAGCATCACTGTG
Isg15 R	GGTGTCCGTGACTAACTCCAT
Oas3 F	TCTGGGGTCGCTAAACATCAC
Oas3 F	GATGACGAGTTCGACATCGGT
Stat1 F	TCACAGTGGTTCGAGCTTCAG
Stat1 R	GCAAACGAGACATCATAGGCA
Mx1 F	CAGAGGTCAGCAGGACATCC
Mx1 R	TCGCTTGCACTCTGATGACT
Ip-10 F	CGTCATTTTCTGCCTCATCCT
Ip-10 R	TGGTCTTAGATTCCGGATTCAG
Il-10 F	TGAATTCCCTGGGTGAGAAGCTGA
Il-10 R	TGGCCTTGTAGACACCTTGGTCTT
EV-A71 VP1-F	GATATCCCACATTCGGTGA
EV-A71 VP1-R	TAGGACACGCTCCATACTCAAG
Gapdh F	CTCTGGAAAGCTGTGGCGTGATG
Gapdh R	ATGCCAGTGAGCTTCCCGTTCAG

Table S2. Antibodies.

Name	Supplier	Cat no.	Experiment
EV-A71 VP1	GeneTex	132339	WB, IF
Anti-APOBEC3G/A3G antibody	abcam	Ab75560	WB
APC/Cyanine7 anti-mouse CD45 Antibody	Biolegend	103116	Flow cytometry
FITC anti-mouse CD3 Antibody	Biolegend	100204	Flow cytometry
PE anti-mouse CD4 Antibody	Biolegend	100407	Flow cytometry
APC anti-mouse CD69 Antibody	Biolegend	104514	Flow cytometry
PE/Cyanine7 anti-mouse CD8a Antibody	Biolegend	100721	Flow cytometry
APC/Cyanine7 anti-mouse CD4 Antibody	Biolegend	100413	Flow cytometry
PE/Cyanine7 anti-mouse NK-1.1 Antibody	Biolegend	108714	Flow cytometry
PE anti-mouse IFN-γ Antibody	Biolegend	505808	Flow cytometry
APC anti-human/mouse Granzyme B Recombinant Antibody	Biolegend	372203	Flow cytometry
FITC anti-mouse/human CD11b Antibody	Biolegend	101206	Flow cytometry
PE anti-mouse/human CD11b Antibody	Biolegend	101207	Flow cytometry
APC/Cyanine7 anti-mouse/human CD11b Antibody	Biolegend	101226	Flow cytometry
FITC anti-mouse Ly-6C Antibody	Biolegend	128006	Flow cytometry
PE anti-mouse CD69 Antibody	Biolegend	104508	Flow cytometry
PE anti-mouse Ly-6C Antibody	Biolegend	128008	Flow cytometry
PE/Cyanine7 anti-mouse F4/80 Antibody	Biolegend	123114	Flow cytometry
APC anti-mouse Ly-6G Antibody	Biolegend	127613	Flow cytometry
PE anti-mouse I-A/I-E Antibody	Biolegend	107608	Flow cytometry
APC anti-mouse CD11c Antibody	Biolegend	117310	Flow cytometry
PE/Cyanine7 anti-mouse CD80 Antibody	Biolegend	104734	Flow cytometry
FITC anti-mouse CD86 Antibody	Biolegend	105005	Flow cytometry
APC anti-mouse CD206 (MMR) Antibody	Biolegend	141708	Flow cytometry
PE/Cyanine7 anti-mouse/human CD45R/B220 Antibody	Biolegend	103221	Flow cytometry
Phospho-STING (Ser365) (D8F4W) Rabbit mAb	Cell Signaling Technology	72971	WB
STING (D2P2F) Rabbit mAb	Cell Signaling Technology	13647	WB
β-Actin (8H10D10) Mouse mAb	Cell Signaling Technology	3700	WB
Phospho-IRF3 (Ser386) (E7J8G) XP Rabbit mAb	Cell Signaling Technology	37829	WB
IRF3 (D614C) XP Rabbit mAb	Cell Signaling Technology	11904	WB
Phospho-TBK1 (Ser172) (D52C2) XP Rabbit mAb	Cell Signaling Technology	5483	WB
TBK1 (E813G) Rabbit mAb	Cell Signaling Technology	38066	WB

Phospho-NF-κB P65 (Ser536) (93H1) Rabbit mAb	Cell Signaling Technology	3033	WB
NF-κB P65 (D14E12) XP Rabbit mAb	Cell Signaling Technology	8242	WB
Phospho-STAT1 (Tyr701) (58D6) Rabbit mAb	Cell Signaling Technology	9167	WB
STAT1 (D1K9Y) Rabbit mAb	Cell Signaling Technology	14994	WB
ISG15 antibody	Cell Signaling Technology	2743	WB
Phospho-STING (Ser365) (D1C4T) Rabbit mAb	Cell Signaling Technology	62912	IF
STING (E9X7F) Rabbit mAb	Cell Signaling Technology	90947	IF
Goat anti-rabbit HRP-labeled Antibody	Cell Signaling Technology	7074	WB
Goat anti-mouse HRP-labeled Antibody	Cell Signaling Technology	91196	WB
